# Gender and the treatment of immune-mediated chronic inflammatory diseases: rheumatoid arthritis, inflammatory bowel disease and psoriasis: an observational study

**DOI:** 10.1186/1741-7015-10-82

**Published:** 2012-08-01

**Authors:** Nienke Lesuis, Ragnar Befrits, Filippa Nyberg, Ronald F van Vollenhoven

**Affiliations:** 1 Medical Faculty, Radboud University, Geert Grooteplein 21, 6500 HB, Nijmegen, The Netherlands; 2 Gastrocentrum Medicin, Karolinska University Hospital, 17176 Stockholm, Sweden; 3 Uppsala University Hospital, Uppsala, and Unit for dermatology, Institution for clinical sciences, Karolinska Institutet, Danderyd hospital, SE-182 88 Stockholm, Sweden; 4 Unit for Clinical Therapy Research Inflammatory Diseases (ClinTRID), The Karolinska Institut. D10:0, 17176 Stockholm, Sweden; 5 Rheumatology Department, Sint Maartenskliniek, Hengstdal 3, 6500 GM, Nijmegen, The Netherlands

## Abstract

**Background:**

Rheumatoid arthritis (RA), inflammatory bowel disease (IBD), and psoriasis are immune-mediated inflammatory diseases with similarities in pathophysiology, and all can be treated with similar biological agents. Previous studies have shown that there are gender differences with regard to disease characteristics in RA and IBD, with women generally having worse scores on pain and quality of life measurements. The relationship is less clear for psoriasis. Because treatment differences between men and women could explain the dissimilarities, we investigated gender differences in the disease characteristics before treatment initiation and in the biologic treatment prescribed.

**Methods:**

Data on patients with RA or IBD were collected from two registries in which patients treated with biologic medication were enrolled. Basic demographic data and disease activity parameters were collected from a time point just before the initiation of the biologic treatment. For patients with psoriasis, the data were taken from the 2010 annual report of the Swedish Psoriasis Register for systemic treatment, which included also non-biologic treatment. For all three diseases, the prescribed treatment and disease characteristics were compared between men and women.

**Results:**

In total, 4493 adult patients were included in the study (1912 with RA, 131 with IBD, and 2450 with psoriasis). Most of the treated patients with RA were women, whereas most of the patients with IBD or psoriasis were men. There were no significant differences between men and women in the choice of biologics. At treatment start, significant gender differences were seen in the subjective disease measurements for both RA and psoriasis, with women having higher (that is, worse) scores than men. No differences in objective measurements were found for RA, but for psoriasis men had higher (that is, worse) scores for objective disease activity measures. A similar trend to RA was seen in IBD.

**Conclusions:**

Women with RA or psoriasis scored significantly higher on subjective, but not on objective, disease activity measures than men, and the same trend was seen in IBD. This indicates that at the same level of treatment, the disease has a greater effect in women. These findings might suggest that in all three diseases, subjective measures are discounted to some extent in the therapeutic decision-making process, which could indicate undertreatment in female patients.

## Background

Rheumatoid arthritis (RA) is a chronic systemic disease that affects the synovial joints. Joint inflammation may lead to progressive destruction of bone and cartilage, which eventually causes loss of function and disability [1]. To control symptoms and prevent joint damage, treatment with disease-modifying antirheumatic drugs (DMARDs) such as methotrexate and sulfasalazine, is the cornerstone of RA management. More recent treatments include biologic drugs of which anti-tumor necrosis factor (anti-TNF) agents are the most frequently used [1,2].

The expression and clinical course of RA are influenced by gender. In developed countries the prevalence of RA is 0,5 to 1.0%, with a male:female ratio of 1:3. The reason for this imbalance is not clear, but both genetic and hormonal factors are thought to be involved. In most studies focusing on the relationship between gender and RA, women were found to have higher disease activity scores, more pain and greater loss of function, both in early and established disease [3-6]. Other studies have reported faster progression of disability in women as measured by the Health Assessment Questionnaire (HAQ), and a lower remission rate in women with early RA [3,7]. Although these studies suggest a less favorable course in women, there is also evidence to the contrary. One study found that erosive disease (that is, severe joint destruction) was more common in men, and also occurred earlier in the disease course. However, women underwent more surgical operations to correct the consequences of joint destruction [8].

Crohn's disease (CD) and ulcerative colitis (UC) are two of the most common forms of inflammatory bowel disease (IBD), a group of chronic systemic diseases causing inflammation of the gastrointestinal tract. UC affects only the large bowel, whereas CD can involve the whole gastrointestinal tract. In the treatment of IBD, corticosteroids, aminosalicylates, and immunosuppressive agents are used to induce and remain long-term remission. When these fail to achieve sufficient disease control, biologic agents can be used, with infliximab being the most widely used biologic in IBD treatment [9]. The literature concerning gender and IBD is relatively limited and to some extent contradictory. The male:female incidence ratio is estimated to be around 1:1.5 for IBD [10]. Female gender was reported to be a risk factor for earlier recurrence of CD after surgery in two studies [10,11], but male gender was found to be a greater risk in another study [12]. Gender was not a prognostic factor for disease course [13]. IBD is associated with an increased risk of colorectal cancer, but this risk seems to be lower in females [14].

Psoriasis encompasses a group of chronic, immune-mediated inflammatory skin and joint diseases, of which chronic plaque psoriasis is the most common form. In the treatment of psoriasis, topical agents are used first, followed by or in combination with phototherapy and/or finally systemic therapy [15]. Both non-biologic (methotrexate, acitretin, cyclosporine and UV therapy) and biologic agents (etanercept, adalimumab, infliximab and ustekinumab) can be used as systemic therapy [16]. There is no difference in the male:female ratio, with an estimated prevalence of 2% for both sexes [17]. A few studies on health -related quality of life (HRQOL) have suggested that with the same degree of skin disease, women experience more stigmatization and worse HRQOL [18,19]; however, a systemic review did not find any relationship between gender and quality of life [20].

Thus, there is some evidence for gender differences in all three of these immune-mediated inflammatory diseases. In previous studies, a number of explanations have been given for these gender differences, including that they are caused by differences in the treatment given to men and women. Therefore, we investigated gender differences in treatment by analyzing disease characteristics just before initiation of biologic therapy in patients with RA, IBD, or psoriasis.

## Methods

Patient consent for data registry and subsequent use in research was obtained from patients at the time of inclusion in all three registries, and local ethics committees approved the use of the data for these studies.

### Patients

For this observational study, adult patients with RA, psoriasis or IBD who were treated with biologic agents (etanercept, adalimumab, infliximab, rituximab, abatacept, golimumab, certolizumab pegol, anakinra, tocilizumab, and ustekinumab) between 1999 and 2010 were included. Only the first prescribed biologic for each patient was considered.

Data on patients with RA or IBD were obtained from two different registries in Sweden: the Stockholm TNF follow-Up Registry (STURE) and the Remicade (infliximab) registry, respectively. In STURE, demographic data, disease characteristics, and evaluations of disease activity are registered for all patients with RA treated with biologics in the Karolinska University Hospital, Stockholm. Disease activity measures are performed at inclusion, at 3, 6 and 12 months after treatment start, and every 6 months thereafter. The Remicade registry is kept at the same hospital and includes patients with IBD treated with infliximab. Data files were extracted from these two registries, and missing measurements were manually retrieved from the hospital patient information system.

Since 2007, data on nearly all patients with psoriasis in Sweden given systemic treatment, including biologic agents, have been kept in a nationwide database called PsoReg. Data from this registry were obtained from the published annual report of 2010 [16].

### Assessments

For patients with RA or IBD, basic demographic data, including gender and disease activity measures were collected at a single time point, just before the initiation of the first biologic therapy; for psoriasis, data were collected at time of inclusion in PsoReg. For RA, disease activity was assessed by the Disease Activity Score with a 28-joint count (DAS28). The DAS28 computes a single score from four separate measurements (tender joint count (TJC), swollen joint count (SJC), patient assessment of global disease activity by visual analog scale (VAS), and erythrocyte sedimentation rate (ESR)) [21]. For the patients who had C-reactive protein (CRP) measured, the DAS28-CRP was also computed, with a high score indicating more active disease. ESR (mm/h) and CRP (mg/l) comprised the acute phase reactants measured. The SJC and TJC, respectively, were counted using the standard set of 28 joints. Experienced general pain was rated by the patients on a VAS of 0 to 100 mm. Both patients and physicians had to rate the global disease activity. Patients used a 100 mm VAS (0 = inactive and 100 = strongly active RA), while the doctors used a six-point scale, ranging from 0 (no activity) to 5 (maximum activity). Functional status was measured using the Swedish version of the Stanford HAQ disability index. The HAQ scores range from 0 to 3, with a higher score indicating a higher level of disability [22]. For a subgroup of patients, HRQOL was measured using the EuroQol five dimensions utility score (EQ-5D), which is summarized in a single number which ranges from 0 (death) to 1 (full health), but negative scores are also possible [23,24].

The disease activity measurements used for IBD were hemoglobin (Hb) level (g/l), ESR, CRP, the Harvey-Bradshaw Index (HBI) and the Short Health Scale (SHS). The HBI is a tool for measuring disease activity, which is completed by both patient (general wellbeing, abdominal pain, and number of liquid stools per day) and physician (based on palpable abdominal mass and presence of complications). The sum of all single scores is used as the outcome measure, and a higher score indicates more active disease [25]. The SHS was used to measure HRQOL. It consists of four questions relating to severity of symptoms, influence of the disease on daily life, concern about the disease, and general wellbeing, which are all answered by the patient using a 100 mm VAS. Scores from all four questions are separate outcomes, and a higher score represents a worse HRQOL [26,27].

For psoriasis, two different measures were used: the Psoriasis Area and Severity Index (PASI) and the Dermatology Life Quality Index (DLQI). The PASI is a clinical measurement that takes into account both the extent and the severity of the skin symptoms; scores range from 0 (no psoriasis activity) to 72 points (maximal psoriasis activity). The DLQI is a 10-item dermatology-specific QOL measurement that assesses the effect of skin disease on a patient's HRQOL over a 7-day period. The total score ranges from 0 to 30, with higher scores indicating worse HRQOL [28]. For both the DLQI and PASI, more than 10 points is considered a high score [16,29]. In the annual report, no distinction was made between the different treatment groups for both PASI and DLQI. Thus the data given in this study apply to the total group of patients with psoriasis treated with systemic medication (that is, the worst cases), which also includes non-biologic systemic treatment. The different parameters are summarized in table 1.

**Table 1:** Parameters for disease activity in rheumatoid arthritis, inflammatory bowel disease and psoriasis

Measurement	Definition
Rheumatoid arthritis	

Swollen joint count (SJC)	Number of swollen joints as determined by the physician out of a standard set of 28 joints

Tender joint count (TJC)	Number of joints tender to palpation (as for SJC)

Patient assessment of pain	Measured by 100 mm visual analog scale (VAS)

Patient assessment of global disease activity	Measured by 100 mm VAS

Physician assessment of global disease activity	Measured on a six-point scale ranging from 'no activity' to 'maximum activity'

Acute phase reactant	ESR (mm/h) and/or CRP (mg/l)

Health Assessment questionnaire (HAQ)	Assessment of functional status (0 to 3 points)

EuroQol five dimensions utility score (EQ-5D)	Assessment of health-related quality of life (HRQOL) (0 to 1)

Disease Activity Score (DAS28)	Assessment of disease activity computed using SJC, TJC, ESR and patient assessment of global disease activity, based on 28 joints. Termed DAS28-CRP when CRP is used instead of ESR

Inflammatory bowel disease	

Hemoglobin level	Measure in g/l

Acute phase reactant	ESR (mm/h) and/or CRP (mg/l)

Harvey Bradshaw Index (HBI)	Assessment of disease activity completed by both patient and physician

Short Health Scale (SHS)	Assessment of HRQOL in four domains, measured by 100 mm VAS

Psoriasis	

Psoriasis Area and Severity Index (PASI)	Assessment of extent and severity of skin lesions consisting of four components; scaling, redness, extent and elevation (0 to 72 points)

Dermatology Life Quality Index (DLQI)	Assessment of the effect of skin disease on HRQOL (0 to 30 points)

ESR, erythrocyte sedimentation rate; CRP, C-reactive protein

### Statistical analysis

Statistical analysis was performed using SPSS statistical software (version 19.0; SPSS Inc., Chicago, IL, USA). To compare differences between men and women, the Mann-Whitney U-test or the independent samples *t*-test was used for continuous variables, and the χ^2 ^test for proportions. For RA and psoriasis, the proportions of men and women prescribed the different biologic drugs were compared within each disease group. Differences in disease characteristics between man and women were evaluated for RA, IBD, and psoriasis separately, and for each biologic in RA. All significance tests were two-tailed and *P*<0.05 was considered significant.

## Results

### Rheumatoid arthritis

In total, 1912 patients with RA (402 men (21%) and 1510 women (79%)) were assessed for the study. They received as a first biologic either a TNF-blocking agent (etanercept, adalimumab, infliximab, certolizumab pegol, or golimumab) or a non-TNF-blocking agent (anakinra, rituximab, abatacept, or tocilizumab). The demographic and clinical characteristics of the cohort are shown in table 2. When biologic treatment was started for the first time in each patient, significant differences between men and women were found for ESR, patient global assessment, TJC, HAQ, DAS28, and DAS28-CRP. All these outcome values were higher in women than in men, with *P*-values ranging between 0.00 and 0.02. By contrast, the physician global assessment and CRP were the same for both genders, and the SJC was numerically, but not significantly, higher in males. General pain scores were somewhat higher in females compared with males, but the difference did not reach significance. There was no difference between genders in age at treatment start, but women had a slightly longer disease duration (*P *= 0.06).

**Table 2:** Demographic and clinical characteristics just before treatment start in patients with rheumatoid arthritis

Parameter	Men	Women	*P *value
Patients, n (%)	402 (21%)	1510 (79%)	

Age at treatment start, years	54.7 (13.7)	54.6 (14.5)	0.93

Disease duration, years	14.7 (10.8)	15.9 (11.2)	0.06

ESR, mm/h	30.1 (21.9)	33.1 (23.3)	0.02^2,3^

CRP, mg/l	25.5 (30.1)	24.9 (31.4)	0.73

Physician global assessment	2.2 (0.8)	2.3 (0.7)	0.38

Patient global assessment	54.3 (24.3)	58.7 (24.1)	0.00*

Pain	54.8 (23.9)	56.9 (24.2)	0.16

Swollen joint count	9.1 (5.9)	8.8 (5.5)	0.36

Tender joint count	7.8 (6.3)	8.7 (6.1)	0.01^3,4^

HAQ	1.13 (0.68)	1.34 (0.68)	0.00^3,4^

DAS28	5.07 (1.37)	5.39 (1.21)	0.00^3,4^

DAS28-CRP	4.87 (1.25)	5.06 (1.14)	0.01^3,4^

EQ-5D	0.49 (0.36)	0.50 (0.30)	0.89

Abbreviations: ESR, erythrocyte sedimentation rate; CRP, C-reactive protein; HAQ, Health Assessment Questionnaire; DAS28, Disease Activity Score using 28 joints; DAS28-CRP, DAS28 using CRP; EQ-5D, EuroQol five dimensions utility score.

^1^Values are mean (SD), except for the first row.

^2^Mann-whitney U-test.

^3^Significant *P *values.

^4^Independent samples *t*-test

Most patients were prescribed a TNF-blocking agent as their first biologic drug, with etanercept, adalimumab, and infliximab being the most frequently prescribed, but no significant differences were seen in the proportions of men and women prescribed any of the nine different biologics (data not shown). Similar percentages of men and women received concurrent therapy with glucocorticoids, non-steroidal anti-inflammatory drugs, or conventional DMARDs. All biologic agents were analyzed separately for gender differences in disease activity parameters; significant differences similar to those in the total group of RA patients were found only for etanercept, adalimumab, and infliximab (data not shown). Gender differences were also analyzed separately for the year in which treatment was started, and a similar pattern to that of the total group of patients with RA was seen (data not shown).

Thus, in RA the female:male proportion of biologics prescribed reflects the overall prevalence of the disease, and objective disease activity parameters were similar between the sexes, but subjective experiences of the disease were significantly worse at this time point in female patients. The compound indices DAS28 and DAS28CRP showed significant differences that were also driven mostly by the subjective components TJC and patient global assessment of disease activity.

### Inflammatory bowel disease

**Figure 1:**
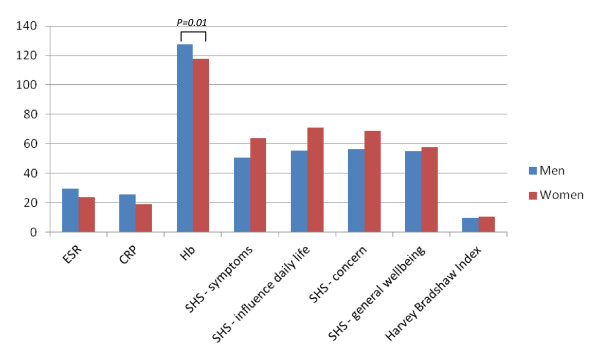
**Clinical characteristics just before treatment start in patients with inflammatory bowel disease (IBD)**. Values are means, and significant values are indicated by brackets. CRP, C-reactive protein; ESR, erythrocyte sedimentation rate; Hb, hemoglobin level; SHS, Short Health Scale.

There were 131 patients with IBD, and all had received infliximab as their first biologic. Most of the patients were men (69.5%), and most had CD (82.4%) or UC (15.3%); the remaining 2.3% of the patients had another form of IBD. Mean age at treatment start was the same for men and women (34.4 versus 35.4 respectively, *P *= 0.71) and there was also no gender difference in disease duration. The clinical characteristics are shown in Figure 1. No significant differences between men and women were found for any parameters. Although the hemoglobin level for men was higher than that for women, this appeared to reflect the physiological sex difference, as there was no gender difference seen in the proportions of men and women having laboratory-defined anemia (data not shown). Male and female patients who were started on biologic treatment had similar values on the HBI, but on three out of four questions of the SHS women had numerically higher scores, although these differences did not reach statistical significance. The disease characteristics were also evaluated for CD and UC separately, which resulted in the same pattern as for the total group (data not shown). Thus, in IBD, both men and women had similar disease severity as measured by the HBI at the start of biologic therapy, but there was a numerical trend suggesting that female patients experience more symptoms.

### Psoriasis

**Figure 2:**
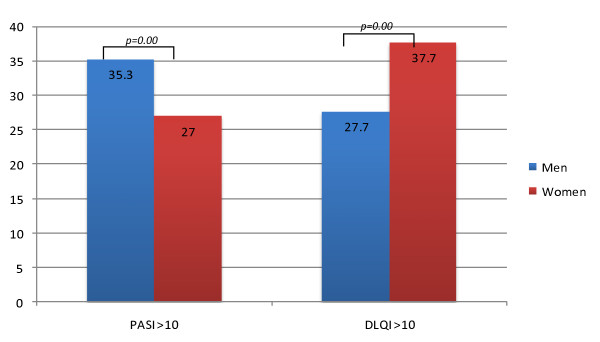
**Proportions of high Psoriasis Area and Severity Index (PASI) and Dermatology Life Quality Index (DLQI) scores in patients with psoriasis**. Values are proportions, and significant values are indicated by brackets.

In total, 2450 patients were enrolled in PsoReg, and all had received a form of systemic treatment, with 589 patients (26.0%) being treated with biologic medications. In both the overall group and the biologic treatment group, most of the patients were men (60.0% and 67.1% respectively). In the whole group, approximately 600 patients (24.5%) were between 31 and 45 years old and 800 (32.7%) between 45 and 60 years; more specific data about mean age or disease duration were not stated in the report. Significantly more men than women had a high score on the PASI, whereas for the DLQI the reverse pattern was seen, with significantly more women having a score of greater than 10 points. The differences are summarized in Figure 2. The same proportions of men and women were prescribed etanercept, adalimumab, and infliximab; only for ustekinumab was a significant gender difference seen (women 7.7%, men 2.5%; *P *= 0.01).

## Discussion

In this study, we investigated gender differences in biologic treatment by examining disease characteristics at the time of biologic initiation in RA, IBD, and psoriasis. We found that there was a treatment difference in both IBD and psoriasis, with men receiving biologic medication more often than women. With regard to the disease characteristics, we found that women with RA or IBD had higher scores than men on subjective but not on objective measures. Only for psoriasis did men score higher on objective disease measurements.

Thus, the greater proportion of men receiving biologic treatment for IBD or psoriasis compared with women was not reflected in the population prevalence rates, which were similar for both sexes in both diseases. This in contrast to RA, for which treatment and population male:female ratios matched each other. For RA, our data confirm earlier studies that did not note any gender differences in the overall use of biologic medications [5,7,30,31]. For both psoriasis and IBD, it had previously been suggested that males are more likely than women to receive systemic treatment (biologic or non-biologic) [32,33], and in the case of psoriasis that female patients are more likely to receive topical treatment whereas men are more likely to receive phototherapy [34].

The gender difference we found in treatment proportions for men and women with IBD or psoriasis gives rise to a number of hypotheses that could explain these findings. Possible reasons that more men than women are treated with biologics include the following: men have more severe disease activity; men may be more likely than women to express treatment preferences for biologics/systemic agents; there may be gender differences in treatment access; the risk:benefit ratio as determined by the physician may be modified by the patient's gender; or there may be a gender difference in the effectiveness of biologicals and of treatment alternatives. With respect to the last point, an interesting point was made by Nyberg *et al*., who suggested that females are more likely to believe that they can influence their disease themselves and therefore tend to assume or be given greater responsibility for their disease (that is, are prescribed more self-care topical treatment) [34]. Later in-depth interviews showed the same tendency [35]. This could mean that through more and better use of an alternative treatment, women with psoriasis need biologic treatment less often than men. Because some patients in this psoriasis cohort were already using biologics at inclusion, similar effects with better adherence to oral medication by women might have influenced PASI scores. However, to our knowledge no reports exist to support this hypothesis. With regard to the risk:benefit ratio, biologic therapies might be considered more dangerous for young women of childbearing age than for men because of possible teratogenic effects, although for biologics this is not yet proven [36,37]. This concern, in combination with age differences, might have been an influence on the discrepant gender ratio for biologic treatment in IBD and psoriasis, as the female patients with RA were on average 20 years older than their IBD and psoriasis counterparts when started on biologics (54.6 for RA versus 35.4 years for IBD and between 20 and 40 years for psoriasis [13]).

With regard to the disease characteristics pertaining before biologic therapy initiation, significant gender differences were found for both RA and psoriasis, and similar numerical differences were found for IBD. At treatment start, female patients with RA had higher values for ESR, patient global assessment, TJC, HAQ, DAS28, and DAS28CRP compared with men. One study on anti-TNF therapy [38] found a similar gender imbalance in objective and subjective measurements as we found in this study. Furthermore, recent data from Arkema *et al*, from the Anti-Rheumatic Therapies in Sweden (ARTIS) registry also support a difference in subjective disease parameters for patients with RA starting biologic therapy.[39] Most studies on gender in RA used time points other than biologic initiation, but the results parallel ours [3-6,40].

Because biologic medications are now used earlier in the disease course than at the time these therapies were first introduced, this could possibly induce gender differences. However, we did not find such evidence, and this is in line with similar studies [39]. The differences in TJC and SJC are not large, they could nonetheless be important when studied at group level. Furthermore, measurements of TJC and SJC in rheumatology practice in the hospital system in which these patients were seen are highly standardized (based on the European League Against Rheumatism handbook) so that interobserver variability is minimized. Another confounder could be a gender difference in the specific joints that are inflamed, but as the individual joints are not recorded in the STURE database, this question remains open.

In psoriasis, a gender difference was found for both the PASI and the DLQI, with men more often having high PASI scores and women more often having high DLQI scores. Our findings for the DLQI parallel a few, but not all, studies on QOL in psoriasis [18-20,41]. For IBD, the SHS, which can be regarded as a short QOL assessment, tended to be higher in women. This matches earlier research in which female gender was found to be a predictor of impaired HRQOL and level of concern [42-44].

Although different assessments were used for the three diseases, a general comparison can be made in that in all three diseases the gender imbalance seems to occur only in subjective measurements such as pain, functional status, and QOL. These results support the hypothesis that the inherent biology of the diseases is similar for both genders, but that females experience their illness as worse, and consequently have a higher symptomatic disease burden. For RA, this is further supported by a study in which women were shown to have more symptoms than men despite similar radiographic joint damage [30]. The higher ESR in female patients with RA could be an exception, but women are known to have higher ESR levels than men, which is most likely due to hormonal factors and a difference in hematocrit concentration [39,45].

The literature regarding gender differences in RA has focused on the possible non-sex neutrality of the disease activity measurements used. For psoriasis and IBD, the same questions may apply. Pain and related measurements are often discussed as being non-sex-neutral. Women are more likely than men to experience different kinds of recurrent pains and also to report more severe levels of pain [46,47]. Moreover, with the same noxious stimuli, women experience more pain than men [48]. These factors can consequently influence pain measurements (TJC, global pain), assessments in which pain is included (DAS28, SHS symptoms, HBI) or assessments that are affected by pain (HAQ, DLQI, SHS). In the case of RA, concomitant fibromyalgia could also influence pain measurements [49].

Another example is the HAQ score, which is known to be higher in females with RA [5,6,30,50]; and this could be attributable to the fact that women have less muscle strength, but it is also possible that men overestimate their functional capacity or that women have higher pain scores [30,48]. Therefore the higher HAQ scores might be caused by the properties of the HAQ rather than by RA; however, it is interesting to note that in the healthy population, no sex differences in HAQ scores are seen [51,52].

Although not formally studied, the PASI might also be influenced by gender. The component of 'scaling' is immediately improved after the use of emollients, and even experienced scorers would probably give a lower score for a skin that has just been treated with emollients. As stated earlier, women are more likely than men to adhere to topical treatment, and consequently, this could mean systematic underscoring of the PASI in female patients.

Thus, it is not clear whether standard disease assessments are sex-neutral, but there is also another way of looking at this question. For example, if women do feel more pain with the same stimulus can it then really be said that the disease is the same? Are fewer compensation resources sufficient reason to disregard a gender difference? It would seem that the subjective disease experience may to some extent be discounted by physicians, whereas for the patient this may be the most important part of their disease.

In summary, we found gender differences in disease characteristics at the start of biologic or systemic treatment in RA, IBD, and psoriasis, with an over-representation of men in the latter two diseases, and greater effect of the disease and worse QOL scores in women with all three diseases. This suggests a potential subtle undertreatment of women with these diseases. We note that undertreatment in RA might then also partly explain the worse outcomes previously reported in longitudinal studies for women with RA.

This study has several limitations, as it was a retrospective observational study with all data having been collected at clinical visits. In addition, it must be noted that the psoriasis results came from an annual report, and were not computed using the raw data. Furthermore, some of the measurements in psoriasis were performed after treatment initiation, and thus the real gender difference might therefore be even bigger. The PASI and DLQI scores were given only for the total group, and not separately for each treatment, therefore subgroup analysis was not possible, thus making comparison with RA and IBD more difficult.

## Conclusion

Women with RA or psoriasis scored significantly higher than men on subjective, but not objective, disease activity measures. A similar trend was seen for IBD. This indicates that all three diseases have a greater effect on women than on men at the same level of treatment. These findings might suggest that in all three diseases, subjective measures are discounted to some extent in the therapeutic decision-making process, which could indicate undertreatment in female patients.

## List of Abbreviations

CRP: C-reactive protein; DAS: Disease Activity Score; DLQI: Dermatology Life Quality Index; EQ-5D: EuroQol five dimensions utility score; ESR: Erythrocyte sedimentation rate; HAQ: Health Assessment Questionnaire; Hb: Hemoglobin; HBI: Harvey Bradshaw Index; HRQOL: Health-related quality of life; IBD: Inflammatory bowel disease; PASI: Psoriasis Area and Severity Index; RA: Rheumatoid arthritis; SHS: Short Health Scale; SJC: Swollen joint count; TJC: Tender joint count; VAS: Visual analog scale

## Competing interests

The authors declare that they have no competing interests.

## List of contributors

NL and RFvV were the major contributors responsible for study design, data analysis, and manuscript writing. RB and FN provided patient data and participated in manuscript writing. All authors had full access to all of the data in the study and can take responsibility for the integrity of the data and the accuracy of the data analysis. All authors have read and approved the final manuscript.

## Pre-publication history

The pre-publication history for this paper can be accessed here:

http://www.biomedcentral.com/1741-7015/10/82/prepub
